# Pulmonary vein isolation through trans-jugular approach in a patient with inferior vena cava interruption

**DOI:** 10.1007/s10840-021-01114-8

**Published:** 2022-01-10

**Authors:** Andrea Saglietto, Gaetano Maria De Ferrari, Federico Ferraris, Matteo Anselmino

**Affiliations:** Division of Cardiology, Department of Medical Sciences, University of Turin, “Città della Salute e della Scienza”, Turin, Italy

## Case report

A 50-year-old man, with surgically corrected congenital heart disease (ostium secundum atrial septal defect associated with partial anomalous pulmonary venous return and inferior vena cava — IVC — interruption), sick sinus syndrome (for which he was implanted with a single-lead atrial pacemaker) and two ablation procedures for right incisional atrial tachycardia, was referred to our institution to undergo pulmonary vein isolation (PVI) due to drug-refractory paroxysmal atrial fibrillation (AF). The rare congenital anomaly (IVC interruption with azygos continuation) precluded conventional inferior transfemoral venous approach; thus, a superior transjugular approach was planned. The procedure was performed under conscious sedation. Transseptal puncture under transesophageal echocardiography guidance was performed via right internal jugular vein with a PREFACE^R^ sheath (Biosense Webster) and a manually curved Brockenbrough needle with a 120° angle to manipulate the tip downward to the fossa ovalis. Electroanatomical (EA) mapping of the left atrium (CARTO, Biosense Webster) was performed using a multipolar catheter (PENTARAY^R^, Biosense Webster). CARTO VIZIGO™ Bi-Directional Guiding Sheath was then introduced and radiofrequency delivered at the pulmonary vein ostia. Complete PVI was confirmed by disappearance of venous potentials on the multipolar mapping catheter, and validated through exit block. No periprocedural complications occurred and the patient was discharged from the hospital in sinus rhythm.

**Fig. 1:**
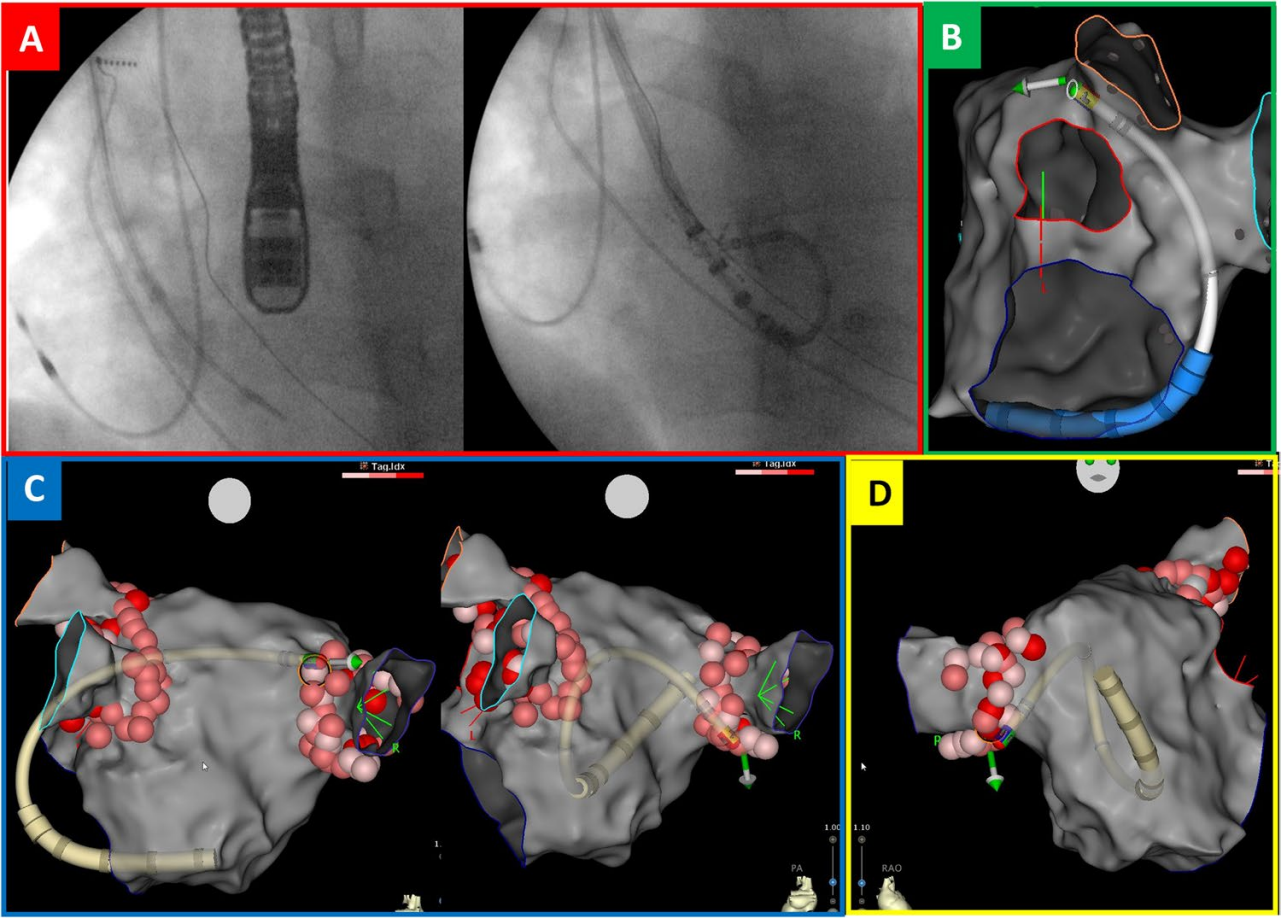
3D visualization of the steerable guiding sheath enabling the ablation catheter to reach target ablation site by unconventional loops. **A** Fluoroscopic anterior views during transseptal puncture (left) and left atrium mapping (right); **B** Latero-lateral 3D EA view during ablation of the ridge between the left superior pulmonary vein and the left appendage; **C** Postero-anterior 3D EA views during right pulmonary vein ablation; **D** Antero-posterior 3D EA view during right pulmonary vein ablation

Left atrial transseptal access is typically performed by inferior transfemoral venous approach. However, in a small subgroup of patients, such as those with congenital IVC interruption, a superior approach from the right internal jugular vein or left axillary/subclavian vein is required to gain access to the left atrium [1–3]. Alternatively, thoracoscopic AF ablation may be considered. In the reported case of AF ablation through unconventional trans-jugular approach, we propose the use of a guiding sheath, visualised on the electroanatomic mapping system, to significantly facilitate ablation catheter manipulation. Radiofrequency application reaching the target site with unconventional loops, such as those shown in Fig. 1 and in the videos (Supplementary Videos 1 and 2), would have hardly been possible without real time, continuous monitoring of the spatial relationship between the steerable guiding sheath and the ablation catheter.

## Supplementary Information

Below is the link to the electronic supplementary material.

Supplementary file1 (MP4 5272 KB)

Supplementary file2 (MP4 49201 KB)

## References

[1] Santangeli P (2020). How to perform left atrial transseptal access and catheter ablation of atrial fibrillation from a superior approach. J Cardiovasc Electrophysiol.

[2] Lim HE (2009). Catheter ablation of atrial fibrillation via superior approach in patients with interruption of the inferior vena cava. Heart Rhythm.

[3] Kato H (2010). Circumferential pulmonary vein ablation of atrial fibrillation via superior vena cava approach in a patient with interruption of the inferior vena cava. Europace.

